# Association study reveals a susceptibility locus with male pattern baldness in the Han Chinese population

**DOI:** 10.3389/fgene.2024.1438375

**Published:** 2024-09-16

**Authors:** Yang Li, He Huang, Bo Liang, Feng-Li Xiao, Fu-Sheng Zhou, Xiao-Dong Zheng, Sen Yang, Xue-Jun Zhang

**Affiliations:** ^1^ Department of Dermatology, The First Affiliated Hospital, Anhui Medical University, Hefei, Anhui, China; ^2^ Key Laboratory of Dermatology (Anhui Medical University), Ministry of Education, Hefei, Anhui, China

**Keywords:** association study, 2q31.1, male pattern baldness, androgenetic alopecia, single nucleotide polymorphisms (SNPs), replication

## Abstract

**Introduction:**

Male pattern baldness (MPB), also known as androgenetic alopecia, represents the most prevalent form of progressive hair loss in humans. It is characterized by a distinctive pattern of hair loss progression from the scalp; however, its underlying mechanism remains elusive and is influenced by hereditary, immune, and environmental factors. Genome-wide association studies (GWASs) have uncovered numerous risk genes/loci among European individuals with MPB. However, the validation of these susceptibility genes/loci within Han Chinese men remains largely unexplored. The aim of this study was to investigate whether the 71 susceptibility loci identified in a recent GWAS among European men also confer risk for MPB in Chinese men.

**Methods:**

Forty-seven single nucleotide polymorphisms (SNPs) previously reported in GWASs of MPB were selected and genotyped in independent individuals comprising 499 Han Chinese cases and 1,489 controls using the Sequenom MassArray system. After stringent quality control measures, 25 SNPs were subjected to statistical analyses. Cochran–Armitage trend test was used to evaluate the association between SNPs and disease susceptibility. To address multiple tests, Bonferroni correction was conducted, setting the threshold for statistical significance at a *p*-value <2 × 10^−3^ (0.05/25).

**Results:**

The rs13405699 SNP located at 2q31.1 exhibited a significant association with MPB in Han Chinese men (*p* = 4.84 × 10^−5^, OR = 1.37, 95% CI: 1.18–1.59). Moreover, the difference in rs13405699 genotype distribution between MPB cases and controls was statistically significant (*p* = 7.00 × 10^−5^). Genotype-based association analysis suggested that the recessive model provided the best fit for the rs13405699 polymorphism.

**Conclusion:**

This study represents the first confirmation of the association between the rs13405699 SNP at 2q31.1 and MPB within the Han Chinese population, thereby enhancing our understanding of the genetic underpinnings of MPB.

## 1 Introduction

Male pattern baldness (MPB), also known as androgenetic alopecia (AGA), is the most prevalent form of progressive hair loss in men. It is commonly characterized by a pattern of hair loss progression on the scalp, with its prevalence varying among different ethnicities. Reports indicate a prevalence of 50% in Caucasian men and 21.3% in Chinese men (Hamilton, 1951; Wang et al., 2010). Although the precise pathogenesis of MPB remains elusive, genetic factors and androgens are crucial for its development (Hamilton, 1951). Genome-wide association studies (GWASs) and meta-analyses have been conducted to elucidate the intricate inheritance patterns of MPB. However, most of these studies have predominantly enrolled European men, whose genetic architecture may differ from that of men belonging to other ethnic backgrounds. Further investigations are warranted to ascertain whether MPB susceptibility genes/loci identified in Europeans also confer risk in Chinese populations.

In August 2013, our initial investigation into known susceptibility loci in people of European descent led to the validation of five single nucleotide polymorphisms (SNPs) at 20p11 associated with AGA in Chinese men (Liang et al., 2013). Subsequently, we conducted a comprehensive search of the most recent and relevant literature published from August 2013 to December 2023 in the PubMed database using the search terms “androgenetic alopecia” or “male pattern baldness” and “association study” to delve deeper into MPB susceptibility loci. Fifteen publications were retrieved, from which three studies describing SNPs in genes associated with MPB susceptibility were selected. The first study is a large GWAS meta-analysis of MPB which identified 63 MPB-associated loci in 22,518 individuals of European ancestry (*p* < 5 × 10^−8^, METAL), albeit without independent replication (Heilmann-Heimbach et al., 2017). The second study represented the largest GWAS of MPB in the European population, comprising 25,662 cases and 17,928 controls, followed by a validation study involving an independent 16,824 cases and 14,288 controls of European ancestry (Pirastu et al., 2017). They replicated 41 loci reported by Heilmann-Heimbach et al. (2017) and identified 30 previously unreported susceptibility loci in a European sample (Pirastu et al., 2017). The third study performed GWAS of AGA involving 494 individuals of Korean descent and identified 13 suggestive AGA-associated loci (*p* < 1.00 × 10^−5^) (Kim et al., 2022). To date, no report has revealed an association of these loci with MPB risk in Han Chinese men. This study sought to evaluate the association of loci recently identified in the GWAS with MPB risk in Chinese men to ascertain whether these loci represent risk factors for MPB in this population.

## 2 Materials and methods

### 2.1 Samples and DNA extraction

A total of 499 cases and 1,489 controls were enrolled in this study. All individuals comprised males of Han Chinese ancestry (Table 1). The hair status of each individual was evaluated by two experienced dermatologists in accordance with the Hamilton ⁄Norwood (HN) classification. The affected men were less than 30 years old with IV–VII of HN grades, or less than 40 years old with V–VII of HN grades. The controls were above 60 years old and showed no signs of AGA or family history of AGA, including first-, second-, and third-degree relatives. Genomic DNA was extracted from peripheral blood lymphocytes using standard procedures of the QIAamp DNA Blood Kit (Qiagen, Valencia, CA, United States). Written informed consent was obtained from all participants. The Institutional Ethics Committee of the First Affiliated Hospital of Anhui Medical University approved the study. The study was conducted according to the principles of the Declaration of Helsinki.

**TABLE 1:** Summary of MPB cases and controls.

Characteristic	Cases^a^	Controls^b^
Total number	499	1,489
Age (years)	28.82 ± 4.94	66.13 ± 5.25

**Note:**

^a^ Affected men were aged <30 years with HN, grades IV–VII, or <40 years with HN, grades V–VII.

^b^ The controls were aged >60 years and showed no signs of MPB.

### 2.2 Single nucleotide polymorphism selection

We selected the SNPs with a *p*-value reaching genome-wide significance (*p* < 5 × 10^−8^), which was reported in GWASs of other ethnicities for replication in Han Chinese men. Among the three recent GWASs, the GWAS by Kim et al. (2022) only presented suggestive associations (*p* < 1.00 × 10^−5^) as the *p*-value did not reach *p* < 5 × 10^−8^; therefore, we excluded these suggestive AGA-associated loci reported by Kim et al. (2022) for further analysis. Meanwhile, the GWAS by Heilmann-Heimbach et al. (2017) is only a meta-analysis of MPB without independent replication. The GWAS by Pirastu et al. (2017) is the largest GWAS of MPB including preliminary screening and a replication stage study, replicating the findings of Heilmann-Heimbach et al. (2017) and identifying several novel loci in the European population. Hence, we selected the loci reported by Pirastu et al. (2017) for further analysis in this study. Pirastu et al. (2017) identified 71 independent loci associated with MPB in a European population (Supplementary Table 1). In total, 12 SNPs were excluded owing to monomorphism, and 12 SNPs were excluded owing to a low minor allele frequency (MAF, <0.05) in Han Chinese in Beijing (CHB) obtained from the 1000 Genomes Project Phase 3 (Supplementary Table 1). Thus, 47 SNPs were selected for analysis. These 47 SNPs represented 47 independent loci.

### 2.3 Genotyping and quality controls

For this, 47 SNPs were genotyped using the Sequenom MassArray platform (Sequenom Inc., San Diego, California, United States) in the Key Laboratory of Dermatology, Ministry of Education, China. We used the MassARRAY Assay Design 3.0 software to design locus-specific polymerase chain reaction (PCR) and detection primers. Following the manufacturer’s instructions, DNA samples were amplified by multiplex PCR reactions, and the PCR products were subsequently used as templates for locus-specific single-base extension reactions. Next, the resulting products were desalted and transferred to a 384-element SpectroCHIP array. Allele detection was conducted using matrix-assisted laser desorption/ionization-time-of-flight (MALDI-TOF) mass spectroscopy. The mass spectrograms were analyzed by the MassARRAYTyper software (Sequenom Inc., San Diego, California, United States). The exclusion criteria for the genotyped SNPs were a call rate of less than 95% and a deviation from Hardy–Weinberg equilibrium (HWE, *p* < 0.05) in the controls. Twenty-five SNPs passed the quality control and were subjected to statistical analysis.

### 2.4 Statistical analysis

#### 2.4.1 Sample size estimation

Pre-study sample size calculations for all SNPs were performed using CaTS-Power Calculator (Skol et al., 2006). The CaTS-Power Calculator program for calculating the sample size provided the power, alpha, and genotype relative risks (odds ratios). We aimed to determine the sample size with 80% power (0.80) and an alpha significance level of 0.05. As 71 SNPs in independent loci have been reported as risk factors for MPB in the European population, we considered that the odds ratios of these SNPs in MPB of the Han Chinese Population were similar to those of the SNPs reported in GWAS for MPB in the European population. Sample sizes were calculated using a case-to-control ratio of 1:1. As shown in Supplementary Table 1, the investigation of certain SNPs requires a large sample size to identify significant associations of these SNPs with MPB. In fact, we used only a small sample size in this study.

#### 2.4.2 Statistical analyses of a replication association study

The SNPs were tested for significant deviation from HWE in the controls and passed the test with *p*-values >0.05. The Cochran–Armitage trend test was performed to evaluate the association between SNPs and MPB susceptibility. Two-tailed tests were conducted to calculate *p*-values. All statistical analyses were conducted using SPSS version 14.0 and PLINK 1.07. Conservatively accounting for multiple testing using Bonferroni correction, the threshold for statistical significance was set at *p* < 2 × 10^−3^ (0.05/25).

## 3 Results

### 3.1 Single nucleotide polymorphism selection for genotyping

We selected 71 SNPs from the largest GWAS published by Pirastu et al. (2017) to identify the risk factors associated with MPB susceptibility in Han Chinese (Supplementary Table 1). In total, 12 SNPs (rs78448052, rs6752754, rs71421546, rs77177529, rs76067940, rs12203592, rs79593277, rs76972608, rs919462, rs17265513, rs68088846, and rs4827528) were excluded owing to monomorphism and 12 SNPs (rs61784834, rs11578119, rs149801367, rs62146540, rs9846246, rs7642536, rs11714208, rs9398803, rs58788673, rs939963, rs12902958, and rs72809171) were excluded owing to a MAF of less than 0.05 in CHB obtained from 1000 Genomes Project Phase 3 (Supplementary Table 1). Thus, 47 SNPs were selected for analysis in the replication study. Of these, 19 SNPs (including rs9719620, rs985546, rs2064251, rs7542354, rs7976269, rs1704529, rs335145, rs12144907, rs9692245, rs12686549, rs79811440, rs2256843, rs10843003, rs844193, rs13021718, rs12752809, rs70993471, rs59995943, and rs7164914) were removed for further analyses as they did not pass the quality control owing to a low call rate. Additionally, three SNPs (rs71530654, rs11087368, and rs111931356) were excluded owing to significant deviations from HWE. The remaining 25 SNPs were further statistically analyzed.

### 3.2 Sequenom genotyping replication

The allele frequencies of the remaining 25 SNPs in Sequenom genotyping replication are shown in Table 2. Only rs13405699 SNP at 2q31.1 was significantly associated with MPB in Han Chinese men [*p* = 4.84 × 10^−5^, odds ratio (OR) = 1.37, 95% confidence interval (CI): 1.18–1.59]. The minor allele A of rs13405699 SNP was a risk allele for MPB in Han Chinese men. None of the other SNPs exceeded the threshold for statistical significance of association (*p* < 2 × 10^−3^) in this study.

**TABLE 2:** Summary of association results of 25 SNPs in nineteen loci between Male pattern baldness cases and controls.

Chr	Gene	SNP	Allele^a^	MAF^b^		P	OR	95%CI
				Case	Control			
1p32.3	*DMRTA2*	rs10888690	C/T	0.1057	0.1023	0.8018	1.03	0.82–1.30
1p34.2	*CITED4*	rs16827770	G/A	0.1194	0.1298	0.3794	0.91	0.73–1.13
2p13.3	*FAM136A*	rs2706768	C/T	0.3420	0.3377	0.8230	1.02	0.88–1.18
2q22.3	*TEX41*	rs10928235	T/A	0.1810	0.1985	0.2374	0.9	0.74–1.08
2q31.1	*CDCA7/SP3*	rs13405699	A/C	0.3697	0.3005	4.84 × 10^−5^	1.37	1.18–1.59
2q35	*WNT10A*	rs7349332	T/C	0.2802	0.2477	0.0402	1.18	1.01–1.39
2q37.3	*TWIST2*	rs11684254	C/G	0.4613	0.4957	0.0560	0.87	0.76–1.00
3q21.3	*KLF15*	rs35892873	T/C	0.3469	0.3431	0.8399	1.02	0.87–1.18
3q25.1	*AADAC*	rs16863765	A/G	0.4242	0.3946	0.1024	1.13	0.98–1.31
4q21.21	*FGF5-PRDM8*	rs7680591	T/A	0.1589	0.1748	0.2564	0.89	0.74–1.09
4q24	*TET2*	rs12509636	C/T	0.0971	0.1077	0.3449	0.89	0.70–1.13
5q33.3	*EBF1-UBLCP1*	rs1422798	G/C	0.2064	0.2319	0.1195	0.86	0.71–1.04
6p24.3	*OFCC1*	rs34624408	A/-	0.3270	0.3175	0.5982	1.04	0.89–1.21
6q21	*LOC105377923*	rs12214131	A/G	0.1539	0.1234	0.0176	1.29	1.05–1.60
10q21.2	*RHOBTB1*	rs2807691	A/G	0.2211	0.2437	0.1515	0.88	0.74–1.05
10q22.3	*C10orf11*	rs11593840	G/A	0.1925	0.1888	0.7981	1.02	0.85–1.23
10q26.13	*METTL10-FAM53B*	rs3781452	C/T	0.4612	0.4251	0.0465	1.16	1.00–1.34
11p11.2	*ALX4*	rs11037975	C/G	0.2276	0.2001	0.0652	1.18	0.99–1.40
12p12.1	*SSPN*	rs7974900	T/C	0.2048	0.1683	0.0104	1.27	1.06–1.53
14q12	*PRKD1*	rs417054	A/C	0.4627	0.4344	0.1169	1.12	0.97–1.30
17q22	*MSI2*	rs17833789	C/A	0.4522	0.4736	0.2373	0.92	0.79–1.06
18q12.3	*SETBP1*	rs8085664	A/C	0.1497	0.1814	0.0212	0.79	0.65–0.97
18q21.33	*BCL2*	rs7226979	C/T	0.4296	0.4473	0.3376	0.93	0.81–1.08
18p11.22	*APCDD1*	rs29073	C/A	0.3607	0.3169	0.0129	1.22	1.04–1.42
Xp22.31	*FAM9B*	rs5934505	C/T	0.2469	0.2328	0.3667	1.08	0.91–1.28

**Note:**

^a^ Allele, minor allele/major allele.

^b^ MAF, minor allele frequency.

Gene, gene harboring the SNP or nearest to the SNP.

The difference in distribution of the rs13405699 genotype between MPB cases and controls was significant in the Sequenom genotyping replication (*p* = 7.00 × 10^−5^, Table 3). Genotype-based association analysis indicated that the recessive model provided the best fit for rs13405699 (*p* = 7.11 × 10^−5^, OR = 1.85, 95% CI: 1.36–2.52).

**TABLE 3:** Distribution of genotypes and genetic model analysis for rs13405699 in cases and controls.

	Cases (n = 499)	Controls (n = 1,489)	P	OR (95% CI)
Genotype				
AA	73 (14.63%)	126 (8.46%)		
CC	203 (40.68%)	720 (48.36%)		
AC	223 (44.69%)	643 (43.18%)	7.00 × 10^−5^	
Recessive model				
AA/(AC + CC)	73/426	126/1,363	7.11 × 10^−5^	1.85 (1.36–2.52)
Dominant model				
AA + AC/CC	296/203	769/720	2.94 × 10^−3^	1.37 (1.11–1.68)

## 4 Discussion

Genome-wide association studies (GWASs) have revealed many genetic-risk loci for MPB. However, the majority of GWASs were conducted in populations of European ancestry, whose genetic architecture may differ from that of men belonging to other ethnic backgrounds. SNP alleles (their frequency) and linkage disequilibrium (LD) may vary greatly between different populations of humans; for example, in 1000 Genomes Project Phase 3 European (CEU) versus Han Chinese of Beijing (CHB) populations. Consequently, a given SNP might have a different frequency or be monomorphic in the second population. For some causal variants of common diseases, ethnic-related heterogeneity yields varying allele frequency and LD patterns such that associations that can be detected in one population may not be detected in other populations (Adeyemo and Rotimi, 2010; Li and Keating, 2014; Lam et al., 2019). Further investigations are warranted to ascertain whether MPB susceptibility genes/loci identified in Europeans also confer risk in Chinese populations.

We first confirmed that rs13405699 SNP (2q31.1) was associated with MPB in Chinese men, which suggested that this locus could be a common risk factor for MPB among different ethnic populations. There are other SNPs that show a tendency of association, such as rs7349332 at WNT10A, rs12214131 at LOC105377923, rs8085664 at SETBP1, rs7974900 at SSPN and rs29073 at APCDD1 (*p* = 0.04, OR = 1.18, *p* = 0.02, OR = 1.29, *p* = 0.02, OR = 0.79, *p* = 0.01, OR = 1.27 and *p* = 0.01, OR = 1.22, respectively). Further studies using larger sample sizes are needed to verify its significance for AGA susceptibility. The associated SNP rs13405699 at 2q31.1 was located in an LD block containing cell division cycle-associated protein 7 (CDCA7) and SP3. To assess the impact of this locus on the genetic risk of MPB, we further explored these two putative susceptibility genes’ potential biological functions. CDCA7 is a DNA-binding protein that functions as a transcriptional regulator (Li et al., 2021). The transforming growth factor-β (TGF-β) signaling pathway could be activated by CDCA7 (Li et al., 2023) and is significantly upregulated in AGA, and participates in AGA pathogenesis (Lu et al., 2016). TGF-β signaling is also an important androgen-inducible pathway (Parrelli et al., 1998; Serruya and Maor, 2021) that promotes AGA development (Inui and Itami, 2011; Sun et al., 2023). TGF-β1 signaling deficiency could prevent perifollicular fibrosis (Yoo et al., 2006; Balık et al., 2021) and catagen progression (Foitzik et al., 2000; Kang et al., 2022) in AGA. Another candidate gene, *SP3*, encodes a transcription factor that induces apoptosis (Ban and Kozar, 2010; Wilson et al., 2010; Zheng et al., 2024). AGA is characterized by a shorter growth (anagen) phase, which is related to increased apoptosis of hair follicle cells (Liu et al., 2021). This finding suggests that SP3 may play an important role in AGA pathogenesis. Taken together, these results indicate that *CDCA7* and *SP3* are plausible susceptibility genes for AGA. However, further fine-mapping and functional analysis studies are warranted to identify the true causal gene(s) in this locus and the pathogenesis of AGA.

This study has several limitations. SNPs were genotyped using the Sequenom MassArray platform, a high-throughput SNP genotyping application that relies on multiplex PCR, single-base primer extension, and detection of alleles using MS. Several genotyped SNPs had a call rate below 95%, which could not pass the quality control. The main reasons for this were primer interactions and inconsistent primer annealing. The application of TaqMan real-time PCR assays could improve these results. Additionally, we could not confirm whether the other SNPs lacked an association with AGA owing to the limited sample size and insufficient data to detect a true association. Future studies should increase the sample size to improve the statistical power to confirm the association between SNPs and AGA.

## 5 Conclusion

This is the first study to demonstrate that the SNP rs13405699 at 2q31.1 was associated with MPB in Han Chinese men, which implied that some susceptibility genes/loci are shared among different ethnic populations. Exploring the full genetic susceptibility factors of MPB in different ethnic populations may help to improve our understanding of MPB pathogenesis.

## Data Availability

The data presented in the study are deposited in the Figshare repository, accession: https://doi.org/10.6084/m9.figshare.26977045.
