# Supplementary material for: Association study reveals a susceptibility locus with male pattern baldness in the Han Chinese population
Source: Front Genet. 2024 Sep 16;15:1438375. doi: 10.3389/fgene.2024.1438375 (PMC11439668; doi:10.3389/fgene.2024.1438375)
Supplement: Supplementary file 1 [file DataSheet1.PDF]

Supplementary Material

Association Study Reveals a Susceptibility Locus with Male Pattern Baldness in the Han Chinese Population

Yang Li, MD[1,2, *], He Huang, MD[1,2], Bo Liang, MD[1,2], Feng-li Xiao,MD[1,2], Fu-sheng Zhou, MD[1,2], Xiao-dong Zheng, PhD [1,2], Sen Yang, MD[1,2], Xue-jun Zhang, MD [1,2]

[1]Department of Dermatology, The First Affiliated Hospital, Anhui Medical University, Hefei, Anhui, China.

[2]Key Laboratory of Dermatology (Anhui Medical University), Ministry of Education, Hefei, Anhui, China.

*** Correspondence:**

Yang Li *

xiaolizier@163.com

Supplementary Table 1 Pre-study calculation of the sample size for 71 SNPs in MPB patients and controls

Locus	SNP	Chr	Gene of interest	Reference	Sample source	OR （obtained from Reference）	Alleles	MAF[a]（EUR)	MAF[a]（CHB)	Total Sample Size Calculation (1：1 case-control ratio)
1	rs7542354	1	DFFA	Pirastu et al., 2017	European	0.81	A/G	0.237	0.325	1000
2	rs2064251	1	SYF2-RUNX3	Pirastu et al., 2017	European	1.17	G/A	0.309	0.180	2500
3	rs16827770	1	CITED4	Pirastu et al., 2017	European	1.07	G/A	0.288	0.155	16000
4	rs61784834	1	FOXD2	Pirastu et al., 2017	European	1.10	T/C	0.432	0.044	—
5	rs10888690	1	DMRTA2	Pirastu et al., 2017	European	0.91	C/T	0.408	0.117	10000
6	rs12752809	1	WARS2	Pirastu et al., 2017	European	1.16	T/C	0.253	0.228	2400
7	rs12144907	1	RPTN-TCHH	Pirastu et al., 2017	European	1.09	G/A	0.229	0.136	10800
8	rs11578119	1	PRRX1	Pirastu et al., 2017	European	1.11	T/C	0.365	0.015	—
9	rs78448052	1	SOX13	Pirastu et al., 2017	European	0.73	T/C	0.021	1.000 (C)	—
10	rs6752754	2	ESPN	Pirastu et al., 2017	European	0.86	G/A	0.142	1.000 (A)	—
11	rs844193	2	LCLAT1	Pirastu et al., 2017	European	1.08	T/A	0.243	0.150	12400

12	rs13021718	2	SRD5A2	Pirastu et al., 2017	European	0.87	A/G	0.146	0.112	4700
13	rs149801367	2	LOC124906010	Pirastu et al., 2017	European	1.04	T/A	0.426	0.024	—
14	rs62146540	2	FBXL12P1	Pirastu et al., 2017	European	0.89	A/C	0.245	0.019	—
15	rs2706768	2	FAM136A	Pirastu et al., 2017	European	1.04	C/T	0.387	0.335	28600
16	rs10928235	2	TEX41	Pirastu et al., 2017	European	1.09	T/A	0.226	0.199	8000
17	rs13405699	2	CDCA7	Pirastu et al., 2017	European	1.45	A/C	0.041	0.301	320
18	rs71421546	2	HOXD3	Pirastu et al., 2017	European	1.27	A/C	0.039	1.000 (C)	—
19	rs7349332	2	WNT10A	Pirastu et al., 2017	European	1.25	T/C	0.115	0.233	1020
20	rs77177529	2	PAX3	Pirastu et al., 2017	European	0.94	T/C	0.130	1.000 (C)	—
21	rs11684254	2	TWIST2	Pirastu et al., 2017	European	1.29	C/G	0.346	0.485	600
22	rs9846246	3	BBX	Pirastu et al., 2017	European	0.92	A/G	0.435	0.019	—
23	rs35892873	3	KLF15	Pirastu et al., 2017	European	0.90	T/C	0.278	0.345	3900
24	rs7642536	3	COPB2	Pirastu et al., 2017	European	1.21	C/T	0.137	0.005	—
25	rs11714208	3	ALPL	Pirastu et al., 2017	European	0.93	G/A	0.345	0.049	—
26	rs16863765	3	AADAC	Pirastu et al., 2017	European	1.15	A/G	0.275	0.393	2100
27	rs7680591	4	FGF5-PRDM8	Pirastu et al., 2017	European	1.13	T/A	0.408	0.170	4400
28	rs12509636	4	TET2	Pirastu et al., 2017	European	0.94	C/T	0.337	0.083	36000
29	rs76067940	4	DKK2	Pirastu et al., 2017	European	0.83	T/C	0.039	1.000 (C)	—
30	rs335145	5	PPIC-PRDM6	Pirastu et al., 2017	European	0.93	G/A	0.275	0.471	6600
31	rs1422798	5	EBF1-UBLCP1	Pirastu et al., 2017	European	0.84	G/C	0.367	0.272	1560
32	rs12203592	6	IRF4	Pirastu et al., 2017	European	1.21	T/C	0.116	1.000 (C)	—
33	rs34624408	6	OFCC1	Pirastu et al., 2017	European	1.16	TA/T	0.432	0.301	2000
34	rs70993471	6	RUNX2	Pirastu et al., 2017	European	0.86	CT/C	0.211	0.278	2100
35	rs12214131	6	LOC105377923	Pirastu et al., 2017	European	1.11	A/G	0.342	0.146	7000
36	rs9398803	6	CENPW	Pirastu et al., 2017	European	0.88	A/G	0.474	0.010	—
37	rs9692245	7	PDGFA	Pirastu et al., 2017	European	0.89	C/T	0.346	0.141	5600

38	rs71530654	7	TWIST1	Pirastu et al., 2017	European	1.26	G/A	0.409	0.354	780
39	rs58788673	7	EPS15P1	Pirastu et al., 2017	European	1.12	CA/-	0.235	0.044	—
40	rs939963	7	AUTS2	Pirastu et al., 2017	European	0.81	G/C	0.455	0.019	—
41	rs9719620	7	MKLN1	Pirastu et al., 2017	European	0.93	T/C	0.459	0.403	6800
42	rs79593277	8	RSPO2	Pirastu et al., 2017	European	2.31	C/G	0.006	1.000 (G)	—
43	rs59995943	8	TRPS1	Pirastu et al., 2017	European	0.85	C/CT	0.419	0.432	1500
44	rs12686549	9	ZNF462	Pirastu et al., 2017	European	1.11	G/A	0.075	0.078	12000
45	rs2807691	10	RHOBTB1	Pirastu et al., 2017	European	1.07	A/G	0.430	0.218	12000
46	rs11593840	10	C10orf11	Pirastu et al., 2017	European	0.88	G/A	0.411	0.194	3400
47	rs3781452	10	METTL10-FAM53B	Pirastu et al., 2017	European	0.88	C/T	0.400	0.422	2300
48	rs79811440	11	LGR4	Pirastu et al., 2017	European	1.16	C/A	0.039	0.097	4700
49	rs11037975	11	ALX4	Pirastu et al., 2017	European	0.90	C/G	0.300	0.248	4600
50	rs7974900	12	SSPN	Pirastu et al., 2017	European	0.88	T/C	0.233	0.204	3500
51	rs10843003	12	PTHLH	Pirastu et al., 2017	European	0.95	G/T	0.190	0.150	28000
52	rs7976269	12	FAR2	Pirastu et al., 2017	European	0.90	A/G	0.202	0.199	5400
53	rs76972608	12	LINC02419	Pirastu et al., 2017	European	0.89	T/A	0.160	1.000 (A)	—
54	rs417054	14	PRKD1	Pirastu et al., 2017	European	0.92	A/C	0.206	0.398	5800
55	rs7164914	15	TCF12	Pirastu et al., 2017	European	0.93	G/A	0.222	0.495	7400
56	rs12902958	15	LOC107984788	Pirastu et al., 2017	European	1.20	A/G	0.073	0.049	—
57	rs111931356	15	CRTC3-IQGAP1	Pirastu et al., 2017	European	1.07	GT/G	0.4526	0.204	13000
58	rs1704529	16	MIR193BHG	Pirastu et al., 2017	European	0.88	C/T	0.340	0.500	2400
59	rs72809171	17	LINC00670	Pirastu et al., 2017	European	1.23	A/G	0.050	0.005	—
60	rs919462	17	**WNT3-MAPT-PLEKHM1**	Pirastu et al., 2017	European	0.79	T/C	0.241	1.000 (C)	—
61	rs17833789	17	MSI2	Pirastu et al., 2017	European	1.12	A/C	0.445	0.495	3000
62	rs29073	18	APCDD1	Pirastu et al., 2017	European	0.94	C/A	0.489	0.291	12000
63	rs8085664	18	SETBP1	Pirastu et al., 2017	European	0.86	A/C	0.262	0.170	2900

64	rs7226979	18	BCL2	Pirastu et al., 2017	European	1.05	C/T	0.452	0.417	17000
65	rs11087368	20	PAX1	Pirastu et al., 2017	European	0.73	G/GT	0.443	0.325	420
66	rs17265513	20	ZHX3-TOP1	Pirastu et al., 2017	European	0.91	C/T	0.195	1.000 (T)	—
67	rs985546	20	TFAP2C	Pirastu et al., 2017	European	1.11	C/T	0.223	0.175	6000
68	rs68088846	21	RUNX1	Pirastu et al., 2017	European	1.16	A/G	0.192	1.000 (G)	—
69	rs2256843	21	UBE2G2	Pirastu et al., 2017	European	1.06	T/A	0.380	0.257	15000
70	rs5934505	23	FAM9B	Pirastu et al., 2017	European	1.10	C/T	0.272	0.206	6400
71	rs4827528	23	AR	Pirastu et al., 2017	European	0.44	A/G	0.151	1.000 (G)	—

Note: [a] Mean minor allele frequency in European (EUR) and Chinese Han from Beijing (CHB) obtained from 1000 Genomes Project Phase 3
